# Development and validation of the early warning system scores ontology

**DOI:** 10.1186/s13326-023-00296-6

**Published:** 2023-09-20

**Authors:** Cilia E. Zayas, Justin M. Whorton, Kevin W. Sexton, Charles D. Mabry, S. Clint Dowland, Mathias Brochhausen

**Affiliations:** 1 Department of Biomedical Informatics, University of Arkansas for Medical Sciences, Little Rock, Arkansas USA; 2 Department of Surgery, College of Medicine, University of Arkansas for Medical Sciences, Little Rock, AR USA; 3 University of Arkansas for Medical Sciences, Institute for Digital Health & Innovation, 4301 West Markham Street, Slot 781, Little Rock, AR 72205 USA; 4 Department of Medical Humanities and Bioethics, University of Arkansas for Medical Sciences, Little Rock, Arkansas USA

**Keywords:** Biomedical ontology, Early warning scores, Track, Trigger systems

## Abstract

**Background:**

Clinical early warning scoring systems, have improved patient outcomes in a range of specializations and global contexts. These systems are used to predict patient deterioration. A multitude of patient-level physiological decompensation data has been made available through the widespread integration of early warning scoring systems within EHRs across national and international health care organizations. These data can be used to promote secondary research. The diversity of early warning scoring systems and various EHR systems is one barrier to secondary analysis of early warning score data. Given that early warning score parameters are varied, this makes it difficult to query across providers and EHR systems. Moreover, mapping and merging the parameters is challenging. We develop and validate the Early Warning System Scores Ontology (EWSSO), representing three commonly used early warning scores: the National Early Warning Score (NEWS), the six-item modified Early Warning Score (MEWS), and the quick Sequential Organ Failure Assessment (qSOFA) to overcome these problems.

**Methods:**

We apply the Software Development Lifecycle Framework—conceived by Winston Boyce in 1970—to model the activities involved in organizing, producing, and evaluating the EWSSO. We also follow OBO Foundry Principles and the principles of best practice for domain ontology design, terms, definitions, and classifications to meet BFO requirements for ontology building.

**Results:**

We developed twenty-nine new classes, reused four classes and four object properties to create the EWSSO. When we queried the data our ontology-based process could differentiate between necessary and unnecessary features for score calculation 100% of the time. Further, our process applied the proper temperature conversions for the early warning score calculator 100% of the time.

**Conclusions:**

Using synthetic datasets, we demonstrate the EWSSO can be used to generate and query health system data on vital signs and provide input to calculate the NEWS, six-item MEWS, and qSOFA. Future work includes extending the EWSSO by introducing additional early warning scores for adult and pediatric patient populations and creating patient profiles that contain clinical, demographic, and outcomes data regarding the patient.

## Introduction

Early detection and response to patient deterioration are critical to avoid adverse events, preventable morbidity, cardiac arrest, unexpected ICU admission, and mortality [1]. Patients often display indicators of clinical deterioration up to 24 h before a significant clinical event arises needing extensive intervention [2]. Failure to manage these deteriorations appropriately leads to negative patient outcomes [3]. However, clinical deterioration may be masked or not obvious to the treatment team [4, 5]. Thus, the patient may deteriorate to the point of instability or even death before this deterioration is noticed and acted upon. Early Warning Scores (EWSs), also known as track and trigger systems, can assist clinicians in recognizing and responding to patient deterioration at an earlier stage of progression and in many cases improve patient care outcomes [6–10].

Since their introduction in the mid-1990s [11], different EWSs have been developed and implemented within different types of electronic health record (EHR) systems and within diverse healthcare settings. Twenty-eight different early warning system scores have been developed and validated in adult patients [12]. Twenty-seven unique pediatric track and trigger systems have been validated in hospitalized children [13]. The proliferation of EWSs within EHR systems across national and international health care institutions has provided a wealth of patient-level physiological decompensation clinical data that can also be used to advance secondary research, such as quality/safety protocols or population health initiatives. One roadblock to secondary analysis of EWS data is the variability of EWSs across multiple providers and multiple EHR systems. This hinders querying across providers and EHR systems, since EWS types use different parameters mapping and merging the parameters is also not always easily possible. We propose to use ontologies to overcome these problems.

In this paper we describe the development and validation of the early warning system scores ontology (EWSSO), representing three commonly used EWSs: the National Early Warning Score (NEWS), the six-item modified Early Warning Score (MEWS), and the quick Sequential Organ Failure Assessment (qSOFA) [14]. In addition, we demonstrate how the EWSSO can be used to generate and query health system data on vital signs and calculate the NEWS, six-item MEWS, and qSOFA using synthetic data. Our goal is to establish a platform that can eventually foster semantic integration of data captured using different EWSs and generate EWS data for the secondary use of clinical data from EHR systems. The ability to semantically integrate EWS data across multiple healthcare settings with the use of ontologies will advance our goal of using real-word data from EHR systems to improve individual-level and population-level health.

## Background

Early Warning Scores, if implemented within the clinical setting, can become an integral part of the healthcare delivery process by assessing the early signs of clinical deterioration of a patient [12, 15, 16]. When EWSs are used as a track and trigger system a patient’s EWSs can either alert the healthcare team to intervene on behalf of the patient or continue to monitor the patient’s condition [12, 17, 18]. Although the validity of EWSs to predict adverse outcomes has been established across different healthcare settings and among diverse patient populations [19–21], these data are not being maximized by healthcare researchers and informaticists to advance secondary research of clinical data and outcomes stored within EHR systems across multiple healthcare settings.

Researchers of individual institutions may be able to extract EWS data, clean it, and append it with patient demographic and clinical characteristics using traditional data cleaning and merging techniques; but integrating data in this format, which may exist as either structured data or free text, can be inefficient and time consuming [22]. This traditional approach to manually transform and append disparate data sources to answer a single research question can be inefficient, because the procedure is not easily reproduced by other researchers, and it does not transform heterogeneous data sources into actionable clinical information. For instance, for a researcher to have confidence in the use of a colleague's transformed dataset to extract clinical information that may be useful to answer a different scientific question, agreement with the meaning of each term used to define a particular feature must exist. Oftentimes, however, the labels or terms used by individual researchers to provide meaning to features within a dataset may differ in subtle but important ways from other researchers’ ideas. Without a standardized data dictionary that is openly accessible and includes terms agreed upon by all members of the research team and is updated overtime, use of such a data source will decrease or worse it continues to be used, but the clinical information derived is questionable. This is where ontologies can help.

Ontologies are formalized representations of the entities in a domain of discourse that are machine interpretable [23]. They are frequently used to achieve semantic integration of biomedical data [24–28]. The lack of semantic integration between different EWSs implemented within different EHR systems across disparate healthcare institutions is currently hampering our ability to query EWS data to identify factors relevant to different EWSs. Our goal is to achieve semantic integration of EWS data to allow easier appending of heterogeneous patient-level and environmental-level data sets to investigate relationships that extend beyond the clinical setting. For instance, examining the correlation between aspects of social determinants, a patient’s EWSs, and negative health outcomes, such as unplanned ICU readmissions, morbidity, and mortality.

Our ontology aims to represent both the structure and planned process of the National Early Warning Score [29], the six-item modified Early Warning Score [30, 31], and the quick Sequential Organ Failure Assessment [32]. The National Early Warning Score system uses inputs from seven vital function measurement datum: respiratory rate; systolic blood pressure; heart rate; Alert, Voice, Pain, Unresponsiveness (AVPU); body temperature; oxygen saturation; and supplemental oxygen use [29]. There are several Modified Early Warning Score systems that are differentiated by the number of physiological parameters used to derive the final score. For the purposes of our study, we will focus on the six-item MEWS, which uses inputs from six vital function measurement datum, respiratory rate, systolic blood pressure, urine output, heart rate, AVPU, and body temperature [31]. The final representation is of the qSOFA, which uses inputs from three vital function measurement datum, the Glasgow coma scale, respiratory rate, and systolic blood pressure [32].

### Materials and methods

#### Ontology development

**Fig. 1:**
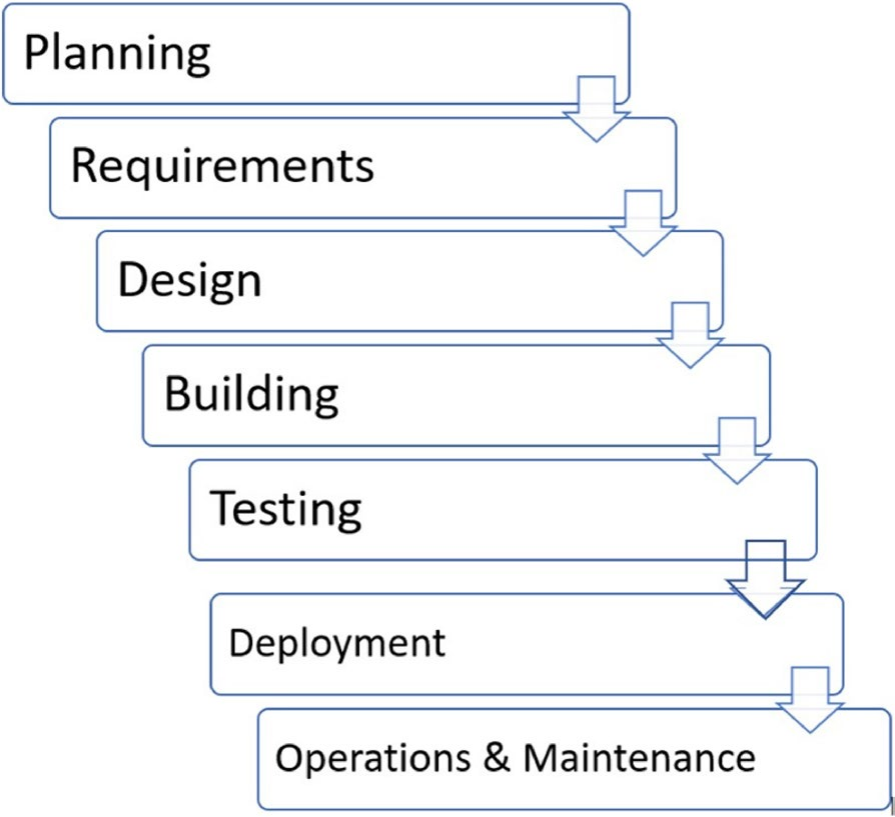
Representation of the seven phases of the SDLC framework

We followed the Software Development Life Cycle (SDLC) framework depicted in Fig. 1 to model the tasks involved in planning, creating, and testing the Early Warning Systems Score Ontology [33]. While EWSSO is available from a public repository, we have not fully gone live with our ontology. Thus, we have not finalized the deployment, operations, and maintenance phases of the SDLC.

### SDLC phase 1 and phase 2: planning and requirements

During the planning phase of the SDLC relevant information and methods to be applied were discussed to assess the feasibility of developing and validating the EWSSO. During the design phase the team agreed to apply methods described by Arp, Smith & Spear for “Building Ontologies with Basic Formal Ontology” [34] particularly, the principles of best practice for ontology design, terms, definitions, and classifications to meet BFO requirements for ontology building. Additionally, we followed Arp, Smith, and Spear’s guidance on using Aristotelian definitions—i.e. genus-differentia definitions—which define an A as a B with the properties C, where A is the class of things being defined (e.g., national early warning score) and B is the immediate superclass (e.g., early warning scores) and C are a set of properties that make the members of A different from the members of B that are not members of A.

As an example, two Aristotelian definitions relevant to our domain are described here.

1. early warning score = def. an information content entity that is about clinical deterioration.
2. national early warning score = def. an early warning score that is the specified output of the national early warning score summation process that has specified input respiratory rate, systolic blood pressure, heart rate, AVPU, body temperature, oxygen saturation, and supplemental oxygen use measurement data.

In the first definition, we specify A [early warning score] as a B [an information content entity] that Cs [is about clinical deterioration]. In the second definition, we specify A [national early warning score] as a B [early warning score] that Cs [is the specified output of the national early warning score summation process that has specified input respiratory rate, systolic blood pressure, heart rate, AVPU, body temperature, oxygen saturation, and supplemental oxygen use measurement data]. If we were to replace the term early warning score in the second sentence with the definition of early warning score in the first sentence the unpacked definition for national early warning score would be an information content entity that is about clinical deterioration and is the specified output of the national early warning score summation process. The result is that we have the same meaning of national early warning score as the sentence with which we began. In keeping in line with creating high-quality ontological definitions we use singular noun terms and avoid acronyms. Aristotelian definitions are helpful for ontology development and maintenance since they bind the definitions to the taxonomy underlying our ontology.

### SDLC phase 3 and phase 4: designing and building

#### OBO foundry principles

The OBO Foundry Principles [35–37] guidelines are meant to serve as the norm for OBO Foundry ontologies and are used in assessing the ontologies that are submitted to the OBO Foundry for review. Because we have plans to submit the EWSSO for the Foundry's assessment, our ontology development process follows these principles. An assessment of how our ontology in the early development phase meets certain OBO Foundry principles is outlined in the results section of this paper. The OBO Foundry aims to be an orthogonal library of interactive ontologies [37]. To prevent conflicting representation and enable optimal semantic integration among resources using OBO Foundry ontologies, a key strategy in developing an ontology for submission to the OBO Foundry is identifying potential areas of overlap with existing OBO Foundry ontologies and prevent conflicting representations [37]. The OBO Foundry principles prescribe the reuse of relations [35], e.g., object properties but considering the reuse of classes is also a common and useful strategy [38]. To address the issue of effective reuse of individual entities or sets of individual entities, OBO Foundry contributors have developed methods of importing classes, object properties, and other entities from existing ontologies [38], called MIREOT. There are multiple implementations of MIREOT. We use the MIREOT Protégé plugin developed by Hanna et al. [39] Reusing entities from preexisting ontologies using MIREOT copies over annotation values from the source ontology. Such annotation values sometimes provide cross-references to other ontologies. E.g., the EWSSO class respiratory rate measurement datum point to the class respiratory rate [40], which in turn points to several similar terms in medical terminologies. These cross-references are coming from an outside source and not subject to our development. Our aim is to follow the strategy to prevent duplicate representation of entities in EWSSO and OBO Foundry ontologies.

#### Reused classes

The 4 reused classes and definitions from OBO Foundry ontologies are listed in Table 1.

**Table 1:** OBO foundry ontologies, reused classes, and definitions

Reused Class	Class Definition	OBO Library Ontology
Process	An occurrent that has temporal proper parts and for some time t, p s-depends on some material entity at t [41]	Basic Formal Ontology (BFO)
Planned process	a process that realizes a plan which is the concretization of a plan specification [42]	Ontology for Biomedical Investigations (OBI)
Information content entity	A generically dependent continuant that is about something [43]	Information Artifact Ontology (IAO)
Measurement datum	An information content entity that is a recording of the output of a measurement such as produced by a device [44]	Information Artifact Ontology (IAO)

#### Reused object properties

We reused four object properties from OBO Foundry ontologies, listed in Table 2.

**Table 2:** OBO Foundry Ontologies, reused object properties, and definitions

Reused object properties	Object property definition	OBO Library Ontology
is about	A (currently) primitive relation that relates an information artifact to an entity [45]	Information Artifact Ontology (IAO)
is_specified_output_of	A relation between a planned process and a continuant participating in that process. The presence of the continuant at the end of the process is explicitly specified in the objective specification which the process realizes the concretization of [46]	Ontology for Biomedical Investigations (OBI)
has_specified_input	The inverse property of is_specified_input_of [47]. Where is_specified_input_of is defined as: “A relation between a planned process and a continuant participating in that process that is not created during the process.” “The presence of the continuant during the process is explicitly specified in the plan specification which the process realizes the concretization of” [48]	Ontology for Biomedical Investigations (OBI)
has_specified_output	The inverse property of is_specified_output_of [49]. Where is_specified_output_of is defined in the second row of this table	Ontology for Biomedical Investigations (OBI)

Every early warning score is an information content entity that is about clinical deterioration. Every early warning score is specified by property is about. The Information Artifact Ontology (IAO) defines object property is about, as “a (currently) primitive relation that relates an information artifact to an entity.” In this case, the early warning score is the information artifact that is about some entity, clinical deterioration, that is “the process of the diminishing realization of the patient's vital functions.” The Ontology for Biomedical Investigations (OBI) defines object property is specified output of as “a relation between a planned process and a continuant participating in that process. The presence of the continuant at the end of the process is explicitly specified in the objective specification which the process realizes the concretization of.” In this case, the national early warning score is the continuant at the end of the national early warning score summation process that is “…explicitly specified in the objective specification which the process realizes the concretization of.”

According to BFO, reality is made up of two types of phenomena: (1) continuants, which are things like objects, qualities, and functions that last over time; and (2) occurrents, which are the activities that continuants engage in [50–52]. Continuants may be independent or dependent [52]. Entities we encounter daily (e.g., person, ball, pencil) are primary examples of independent continuants that also act as carriers of dependent continuants (e.g., a smile, the color of a ball, the ability to write) [52]. We mention these BFO fundamental concepts of reality because we classify early warning system scores, such as the NEWS, six-item MEWS, and qSOFA as generically dependent continuants, more specifically an information content entity, that are the specified output of a planned process (occurrent) that uses attribute specific measurement inputs to calculate a patient’s early warning score, which warns about clinical deterioration.

We created our ontology in OWL2 using the Protégé ontology editor [53] and provided visualization of our ontology using the online MIRO software [54]

### SDLC phase 5: testing

To determine the usefulness of our ontology to generate and query heterogeneous vital sign data for early warning score calculation and secondary data analysis we performed the following steps.

#### Step 1: create synthetic datasets

The purpose for creating synthetic datasets was to simulate heterogeneous data sources of critical care patients whose degree of physiological decompensation is typically evaluated with an early warning system score, such as the NEWS, MEWS, and / or qSOFA. We created two different synthetic datasets. The first included vital sign measurement features specific to each of our early warning scores. The second dataset included other clinical and demographic features, unnecessary for early warning score calculator. This was done to test whether our process with ontological foundation could differentiate between features necessary and unnecessary for score calculation. Temperature measurement data were added as either degree Fahrenheit or degree Celsius. This was done so that the system would then calculate the early warning score with the appropriate temperature measurement scale. Additionally, a random number of values were left blank to represent missingness, common to all datasets. Our process is not meant to impute missing data. However, if a feature necessary to calculate an early warning score is missing the system will return the output as unavailable. Each dataset included 1,000 patient rows.

To create our synthetic datasets of vital measurements used in various early warning scores, we first needed a method to generate a normal distribution of values with a set median, interquartile range (IQR), and extreme range. Using a JavaScript example as a base [55] a Python script was created to generate N samples with configurable parameters (median, IQR, and range) for each vital measurement needed. For the dataset to prove useful in a comparison of early warning scores, we needed to base the vitals on a set of data pertaining to emergency / critical care. We used a Danish study by Nissen et. al. [56] of the performance of NEWS as the baseline parameters to determine most of our measurements since the Danish Multicenter Cohort focused on emergency department admissions. These patients would have distribution and ranges of EWS variables that are more indicative of the types of patients who would be subject to these types of early warning scores for deterioration assessment. The listed median and IQR for each measurement were used in conjunction with a set of extreme ranges that ensured the full range of NEWS scoring would be represented [56]. Any missing measurements were filled from similar studies regarding critical patients in European cohorts [16]. To better align the results of different early warning scores using the distinct but related measurements for consciousness, AVPU vs GCS, we generated the score for AVPU and then converted to GCS per Romanelli and Farrell [57].

#### Step 2: convert synthetic dataset into triples

The synthetic datasets were converted into triples in an RDF database informed by our early warning system scores ontology. We transformed the entirety of each dataset to triples.

#### Step 3: visualization of RDF database and SPARQL queries

**Fig. 2:**
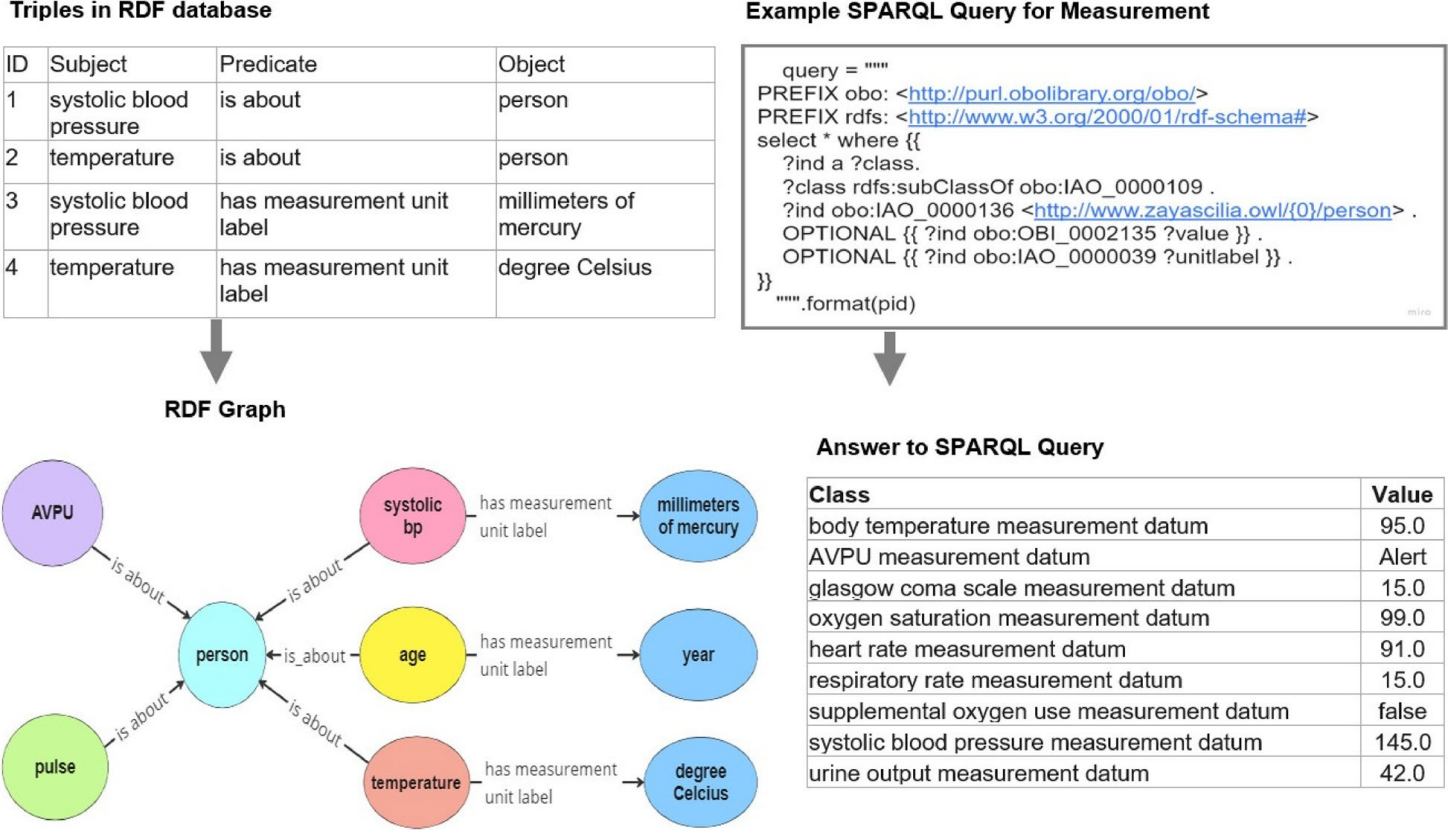
Representation of RDF database

Once the heterogeneous synthetic datasets were converted into triples representing each patient and their respective vital sign measurements, the results were loaded into GraphDB. We then ran a SPARQL query to GraphDB that returned all measurement data for a specific patient (patient identifier). A visualization of the triples in the RDF database, RDF graph, SPARQL query, and the answer for the SPARQL query used to extract a person’s measurement data can be seen in Fig. 2.

#### Step 4: calculate the early scores for each person

To calculate the early warning scores for each person, three projects were created in Python. The intention was to define the required calculations in separate modules that would be imported and utilized by the base project. One project defined each early warning score’s requirements and the formula for the score calculation. We used the guide charts for NEWS, six-item MEWS, and qSOFA scoring system methodology provided by Aseptika Limited [58], the Institute for Healthcare Improvement [59], and Omni Calculator [60], respectively. Another project contained formulas for unit conversions (e.g., converting temperature to degrees Celsius when a formula requires Celsius, but the provided data is Fahrenheit). The general flow of the application was that the base Python project would query the data from the triplestore using SPARQL, representing a person with their semantically tagged measurements. The EWS-specific module would be dynamically imported by the base project and used to calculate each early warning score that meets the minimum data requirements for the score. The calculation is possible because the data representing the measurements is semantically tagged, and the formulas for each score use those tags to determine the correct parameters. In turn, the EWS module would import the unit conversion module to perform any necessary unit conversions on the data. This approach allows new early warning scores or unit conversion formulas to be added to their respective projects without interfering with the base project or other projects created to take advantage of the centralized specification.

#### Step 5: validate early warning system score output

Researcher CEZ randomly selected ten patients and manually calculated the early warning system scores, based on the heterogeneous datasets, and compared to system output.

## Results

### Ontology development

#### OBO foundry principles

The list below indicates how the early-stage development of the EWSSO fulfills several of the OBO Foundry Principles.

- P1) Open. Our ontology is openly available to all users under the Creative Commons CC-BY 3.0 license. This means it may be shared and adopted if appropriately credited and changes, if made, are indicated.
- P2) Common Format. Our ontology is made available in the OWL Web Ontology Language and the Resource Description Framework (RDF).
- P5) Scope. The domain of the ontology is early warning system scores for clinical deterioration in inpatient populations. We start with the simplest and most easily defined features of our relevant domain and will work our way outward to more complex features in future updates of our ontology.
- P6) Textual Definitions. All newly created classes have a textual definition (see Table 3).
- P7) Relations. Relations reused from the Relations Ontology (RO), Basic Formal Ontology (BFO), Ontology for Biomedical Investigations (OBI), and Information Artifact Ontology (IAO).

**Table 3:** New classes and definitions

New Class	Our Definition
Early warning score	An information content entity that is about clinical deterioration [17]
Clinical deterioration	The process of the diminishing realization of the patient's vital functions [61]
National early warning score	An early warning score that is the specified output of the national early warning score summation process that has specified input respiratory rate, systolic blood pressure, heart rate, AVPU, body temperature, oxygen saturation, and supplemental oxygen use measurement data [29]
Six-item modified early warning score	An early warning score that is the specified output of the six-item modified early warning score summation process that has specified input respiratory rate, blood pressure, urine output, heart rate, AVPU, and body temperature measurement data [30]
Quick sequential organ failure assessment	An early warning score that is the specified output of the quick sequential organ failure assessment summation process that has specified input Glasgow coma scale, respiratory rate, and blood pressure measurement data [32]
Alert, Voice, Pain, Unresponsiveness (AVPU) measurement datum	A measurement datum that is the specified output of a process that uses a standardized assessment algorithm to determine a patient's level of consciousness [62]
Glasgow coma scale measurement datum	A measurement datum that is the specified output of a process that uses a standardized rating scale to evaluate the level of consciousness and overall status of the central nervous system [63]
Respiratory rate measurement datum	A measurement datum of the rate at which an organism breathes [40]
Oxygen saturation measurement datum	The measurement datum of the ratio of oxygenated hemoglobin to total hemoglobin in the blood [64]
Body temperature measurement datum	A measurement datum of the temperature of a part of the human body [65]
Systolic blood pressure measurement datum	A measurement datum of the maximum pressure exerted into the systemic arterial circulation during the contraction of the left ventricle of the heart [66]
Heart rate measurement datum	A measurement datum of the number of heartbeats per unit of time, usually expressed as beats per minute [67]
Urine output measurement datum	A measurement datum of a patient's urine output over a specified period
Supplemental oxygen use measurement datum	A measurement datum of a patient's use of supplemental oxygen
Alert, Voice, Pain, Unresponsiveness (AVPU) measurement datum score	An assay that is the specified output of a scoring process that has specified input AVPU measurement datum
Glasgow coma scale measurement datum score	An assay that is the specified output of a scoring process that has specified input Glasgow coma scale measurement datum
Respiratory rate measurement datum score	An assay that is the specified output of a scoring process that has specified input respiratory rate measurement datum
Oxygen saturation measurement datum score	An assay that is the specified output of a scoring process that has specified input oxygen saturation measurement datum
Body temperature measurement datum score	An assay that is the specified output of a scoring process that has specified input body temperature measurement datum
Systolic blood pressure measurement datum score	An assay that is the specified output of a scoring process that has specified input systolic blood pressure measurement datum
Heart rate measurement datum score	An assay that is the specified output of a scoring process that has specified input heart rate measurement datum
Urine output measurement datum score	An assay that is the specified output of a scoring process that has specified input urine output measurement datum
Supplemental oxygen use measurement datum score	An assay that is the specified output of a scoring process that has specified input supplemental oxygen use measurement datum
National early warning score scoring process	A planned process that assigns a score to seven measurement datum based on a predefined rubric and has specified output respiratory rate measurement datum score, systolic blood pressure measurement datum score, heart rate measurement datum score, AVPU measurement datum score, body temperature measurement datum score, oxygen saturation measurement datum score, and supplemental oxygen use measurement datum score
Six-item modified early warning score scoring process	A planned process that assigns a score to six measurement datum based on a predefined rubric and has specified output respiratory rate measurement datum score, systolic blood pressure measurement data score, urine output measurement datum score, heart rate measurement datum score, AVPU measurement datum score, and body temperature measurement datum score
Quick sequential organ failure assessment scoring process	A planned process that assigns a score to three measurement datum based on a predefined rubric and has specified output Glasgow coma scale measurement datum score, respiratory rate measurement datum score, and systolic blood pressure measurement data score
National early warning score summation process	A planned process that provides the sum of seven measurement datum scores, respiratory rate measurement datum score, systolic blood pressure measurement datum score, heart rate measurement datum score, AVPU measurement datum score, body temperature measurement datum score, oxygen saturation measurement datum score, and supplemental oxygen use measurement datum score
Six-item modified early warning score summation process	A planned process that provides the sum of six measurement datum score, respiratory rate measurement datum score, systolic blood pressure measurement data score, urine output measurement datum score, heart rate measurement datum score, AVPU measurement datum score, and body temperature measurement datum score
Quick sequential organ failure assessment score summation process	A planned process that provides the sum of three measurement datum score, Glasgow coma scale measurement datum score, respiratory rate measurement datum score, and systolic blood pressure measurement data score

Our long-term goal is to submit the EWSSO to the OBO Foundry. Hence, the GitHub repository is created following OBO Foundry practices and rules. The repository is modeled to be similar to GitHub repositories, such as OBI [https://github.com/obi-ontology/obi] or OBIB [https://github.com/biobanking/biobanking] among others.

EWSSO is freely available on the GitHub https://github.com/zcilia/ews-ontology.

The data for our experiment can be found here: https://github.com/EWSSO/documentation

- Ontosheep (application full instructions): https://github.com/jmwhorton/ontosheep/tree/main
- Ontosheep (container instructions): https://github.com/jmwhorton/ontosheep/tree/main/test-deploy
- Ontosheep modules (These are the two 'helper' modules for Ontosheep. Modules in separate projects for reusabiity.): https://github.com/jmwhorton/ontosheep-ews and https://github.com/jmwhorton/ontosheep-conversion

#### Newly created classes

Table 3 lists the 29 new classes created and their respective definitions. We also include references reviewed that inspired our new definitions, or the Internationalized Resource Identifier (IRI) of the OBO Foundry ontology classes that we based our new definitions on. 

#### Representation of early warning system score ontology

**Fig. 3:**
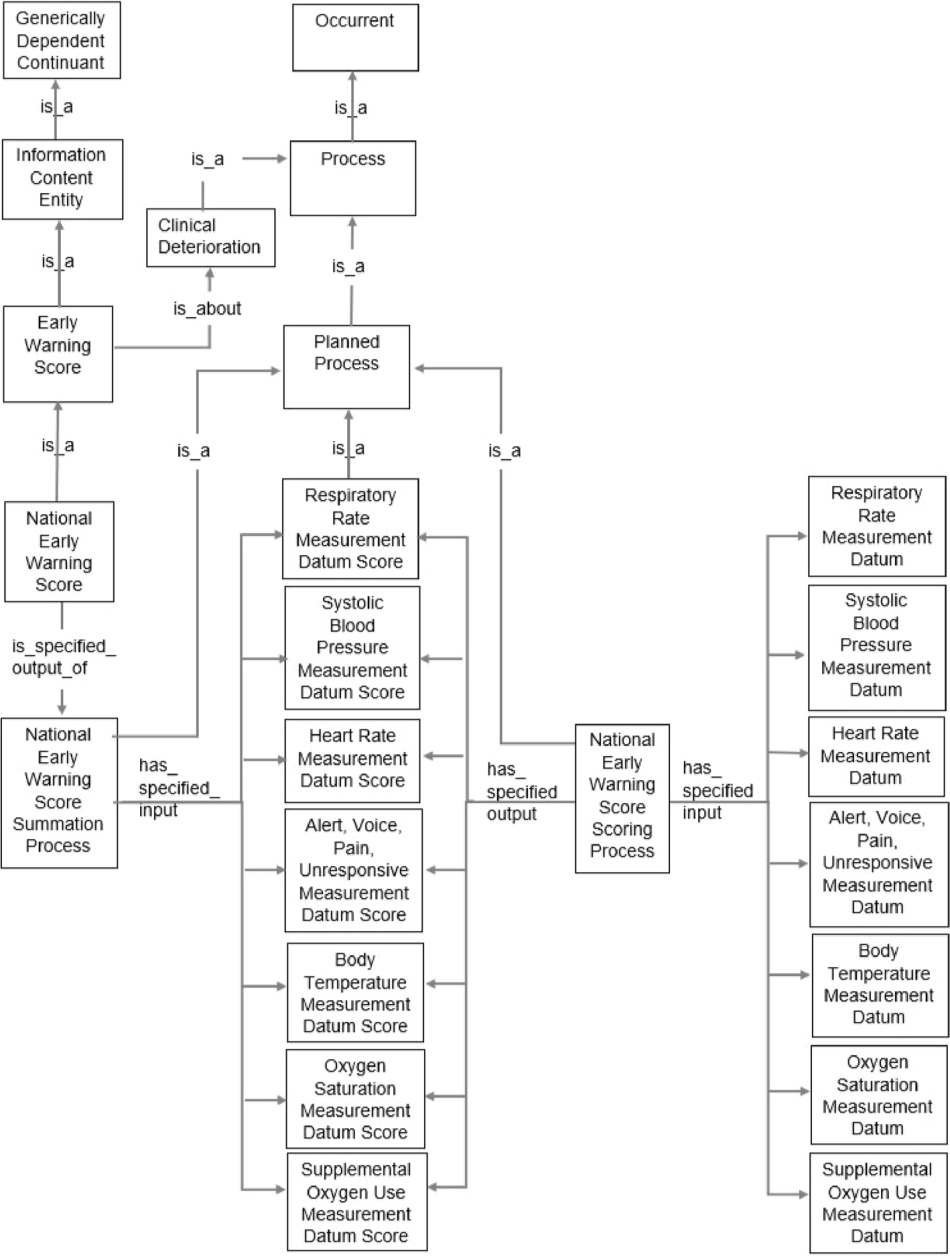
Early warning system score ontology of the national early warning score

**Fig. 4:**
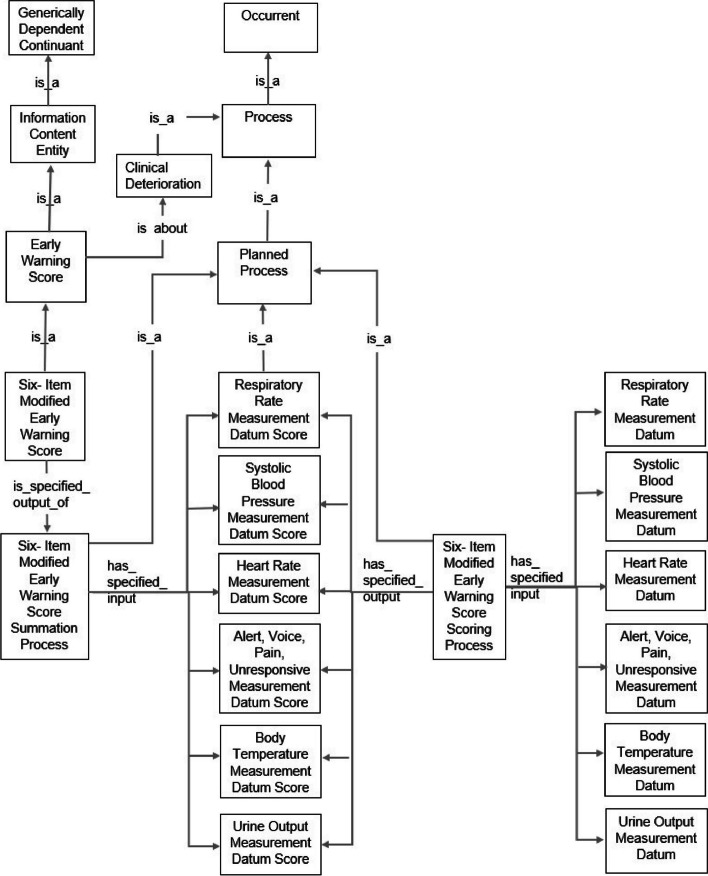
Early warning system score ontology of the six-item modified early warning score

**Fig. 5:**
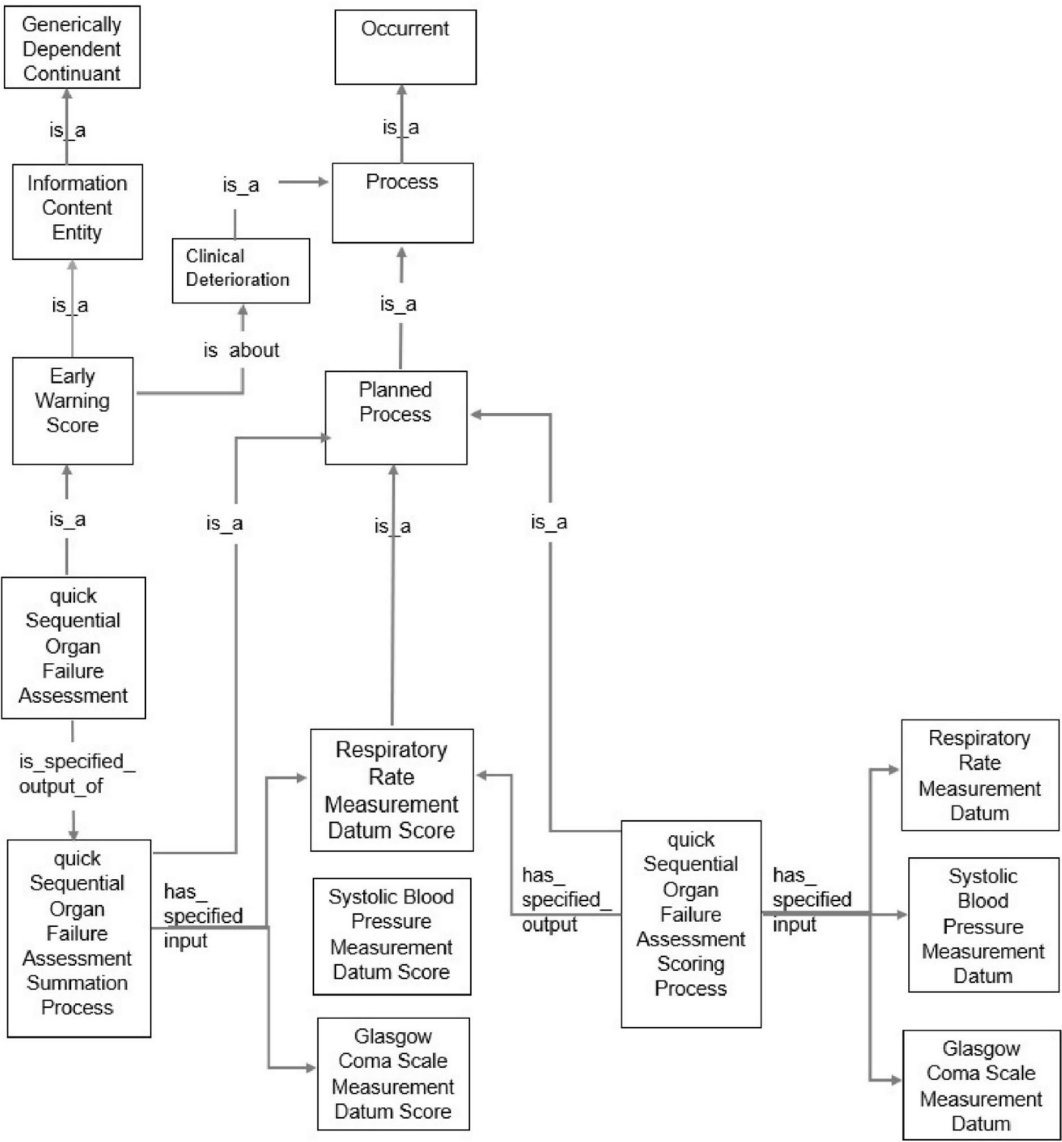
Early warning system score ontology of the quick sequential organ failure assessment

The images of the early warning system score ontology in Figs. 3, 4, and 5 represent the national early warning score, six-item MEWS and qSOFA.

#### Ontology performance evaluation

Our evaluation of EWSSO was to generate and query health systems data and correctly retrieve the data elements required to calculate the NEWS, six-item MEWS, and qSOFA and no other data elements. To test performance, we created two synthetic datasets with 1,000 patient rows each. The first synthetic dataset included vital sign measurement features specific to each of our early warning scores. The second synthetic dataset included other clinical and demographic features, unnecessary for early warning score calculator. Each time we queried the data, our ontology-based process could differentiate between features necessary and unnecessary for score calculation.

## Discussion

One of the core terms for early warning scores is clinical deterioration. We checked multiple ontology lookup resources, e.g., OntoBee [68] and the NCBO Bioportal [69] for a term to re-use. The only term we could find was from Medical Subject Headings (MeSH) resource and defines clinical deterioration as “A critical disease progression, often measured by a set of clinical parameters, which activates HOSPITAL RAPID RESPONSE TEAM” [70]. This definition is not optimal, since it defines clinical deterioration by the activation of a hospital rapid response team. The authors hold that clinical deterioration may exist without a hospital rapid response team present and activated. A recent concept analysis of clinical deterioration did not result in a definition referring to the activation of a hospital rapid response team or any other healthcare procedure [61]. We propose to define clinical deterioration as the process of the diminishing realization of the patient’s vital functions.

While the current project is of limited scope, as only NEWS, MEWS, and qSOFA are considered it serves as the basis for future work with all early warning scoring systems. As the project is consolidated, we are going to seek to integrate additional early warning scores. Although the EWSS ontology does not perform mathematical calculations, it is expected to help users query relevant ontological relationships and measurement data. Using Microsoft Excel, SAS, or other computer programming languages researchers can use the queried data to calculate the early warning scores for secondary analyses of clinical data.

It is our plan to create a pipeline that allows clinical service providers and clinical researchers to perform data acquisition and EWS calculation without the need to run SPARQL queries or write Python commands. During the development of this pipeline, we will recruit a small number of independent users to test it and provide feedback on its usability. During these trials EWSSO will begin to fulfill the independent user requirement of the OBO Foundry. Once EWSSO fulfills all criteria for applying for an OBO Foundry namespace we will apply for a namespace—preferably “EWSSO”—assigned by the OBO Foundry.

## Conclusion

The objective of this paper was to describe the development and validation of the early warning system scores ontology using the principles of best practice for domain ontology design, terms, definitions, and classification as described by Arp, Smith & Spear Building Ontologies with Basic Formal Ontology [14] and aiming to follow OBO Foundry principles [35–37]. We have developed the EWSS Ontology covering three commonly used early warning scores in clinical practice: NEWS, six-item MEWS, and qSOFA. We demonstrated that EWSSO provides the functionality to facilitate search and extraction of data necessary to calculate the scores.

Further work includes extending the EWSS Ontology by introducing additional early warning scores for adult and pediatric patient populations and creating patient profiles which contain clinical, demographic, and outcomes data regarding the patient. The early warning system scores ontology can be implemented and personalized for a particular healthcare system by adding classes for organizational characteristics including clinical staff, non-clinical staff, and management staff. Users of the ontology can develop queries based on the semantically integrated data created, thereby reducing the inefficiency involved with compiling voluminous datasets of heterogeneous EHR data for research or quality improvement initiatives.

## Data Availability

EWSSO is freely available on the GitHub https://github.com/zcilia/ews-ontology. The data for our experiment can be found here:https://github.com/EWSSO/documentation. • Ontosheep (application full instructions):https://github.com/jmwhorton/ontosheep/tree/main. • Ontosheep (container instructions):https://github.com/jmwhorton/ontosheep/tree/main/test-deploy. • Ontosheep modules (These are the two 'helper' modules for Ontosheep. Modules in separate projects for reusabiity.):https://github.com/jmwhorton/ontosheep-ews and https://github.com/jmwhorton/ontosheep-conversion.

## Abbreviations

BFO: Basic Formal Ontology
EWSSO: Early Warning Systems Score Ontology
IAO: Information Artifact Ontology
MEWS: Modified Early Warning Score
NEWS: National Early Warning Score
OBI: Ontology for Biomedical Investigations
qSOFA: Quick Sepsis Related Organ Failure Assessment
